# Adrenal Vein Sampling to Distinguish Between Unilateral and Bilateral Primary Hyperaldosteronism: To ACTH Stimulate or Not?

**DOI:** 10.3390/jcm9051447

**Published:** 2020-05-13

**Authors:** Tae-Yon Sung, Wilson Mawutor Alobuia, Monica Varun Tyagi, Chandrayee Ghosh, Electron Kebebew

**Affiliations:** 1 Department of Surgery, Asan Medical Center, University of Ulsan College of Medicine, Seoul 05505, Korea; 2 Department of Surgery and Stanford Cancer Institute, Stanford University School of Medicine, Stanford, CA 94305, USA; walobuia@stanford.edu (W.M.A.); mtyagi@stanford.edu (M.V.T.); cghosh@stanford.edu (C.G.); kebebew@stanford.edu (E.K.)

**Keywords:** primary hyperaldosteronism, adrenocorticotropic hormone stimulation, selectivity index, lateralization index

## Abstract

The aim of this study is to determine the accuracy of adrenal vein sampling (AVS) with and without adrenocorticotropic hormone (ACTH) stimulation to distinguish between unilateral and bilateral primary hyperaldosteronism (PA). Retrospective analysis of a prospective database from a referral center between 1984 and 2009, 76 patients had simultaneous cannulation of bilateral adrenal veins and AVS with and without ACTH stimulation. All patients had adrenalectomies. The selectivity index (SI, cut-off value ≥2) was used for confirmation of successful cannulation of the adrenal vein. The lateralization index (LI, cut-off value >2 and >4) was used for distinguishing between unilateral and bilateral PA. The SI ratio was higher with ACTH stimulation compared to without for the right adrenal vein (*p* = 0.027). The LI > 2 ratio was higher with ACTH stimulation compared to without (*p* = 0.007). For the LI > 4 ratio, there was no difference between with and without ACTH stimulation (*p* = 0.239). However, for a LI > 4, 7 patients (9.2%) were not lateralized with ACTH stimulation, but they did lateralize without ACTH stimulation. AVS with ACTH stimulation is associated with a higher SI ratio compared to AVS without ACTH stimulation. However, when using LI > 4 for AVS, samples without ACTH stimulation should also be included to detect a subset of patients with unilateral disease that are not detected with ACTH stimulation.

## 1. Introduction

Primary hyperaldosteronism (PA) results from excessive aldosterone production from the adrenal cortex and it affects 4.3%–10% of general hypertensive patients [1,2] and up to 20% of patients with resistant hypertension [3]. PA is the most common cause of secondary hypertension, and it is one of the surgically curable causes of hypertension [4]. PA may be due to unilateral or bilateral hypersecretion of aldosterone. Differentiating unilateral from bilateral disease is important because patients who have unilateral PA can be cured with unilateral adrenalectomy, while medical treatment is recommended for bilateral PA [2,5,6].

Adrenal vein sampling (AVS) is recommended to distinguish between unilateral and bilateral PA before adrenalectomy in patients older than 35 years old, and in patients younger than 35 years with bilateral normal adrenal glands or bilateral adrenal nodules [6,7,8,9,10,11,12]. Some centers prefer the selective use of AVS only when preoperative anatomic imaging cannot definitively lateralize the aldosteronoma (patients with bilateral normal adrenal glands or bilateral adrenal masses), because successful catheterization of both the right and left adrenal veins is technically challenging and may be associated with complications [11,13]. Some centers prefer not to perform AVS in older individuals with bilateral adrenal abnormalities and use medical therapy. However, more centers are recommending AVS to determine whether the patient is a surgical candidate, even if computed tomography (CT) imaging shows unilateral masses and a normal contralateral adrenal gland [2,6,8,14].

AVS can be performed with or without adrenocorticotropic hormone (ACTH) stimulation [2,8,15,16,17,18]. Many referral centers around the world perform ACTH stimulation to avoid the problem of aldosterone and cortisol pulsatile variation and increase the gradient as compared to non-stimulation [15,19]. However, there are no specific guideline recommendations as to whether ACTH stimulation is necessary for AVS or improves the ability to distinguish between unilateral and bilateral PA [2,7,17]. In this study, with simultaneous cannulation of the bilateral adrenal veins, we determined the accuracy of AVS with and without ACTH stimulation in the same patients to distinguish between unilateral and bilateral PA.

## 2. Materials and Methods

### 2.1. Patients

**Figure 1:**
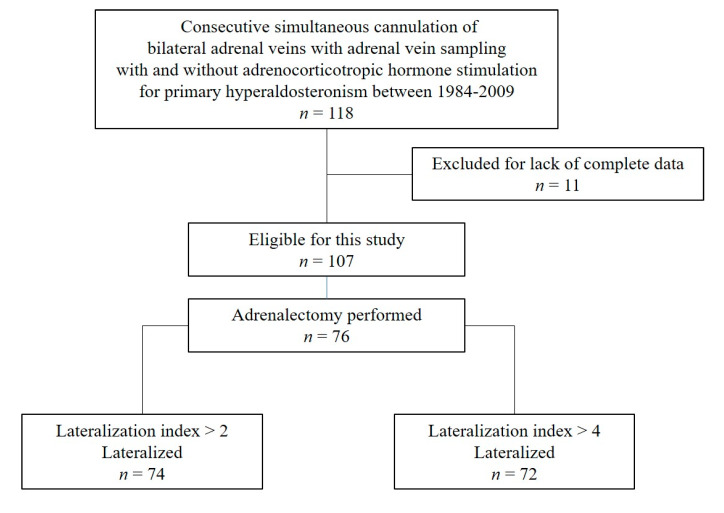
Selection of the study population.

We performed a retrospective analysis of PA patients who had AVS between 1984 and 2009. The study was conducted at Stanford University. Patients had simultaneous cannulation of bilateral adrenal veins and had AVS with and without (before ACTH infusion) ACTH stimulation. A total of 118 consecutive patients had simultaneous cannulation and had AVS with and without ACTH stimulation during the study period. Among them, 11 were excluded because of the lack of complete data. Of the 107 patients with complete data, we included 76 patients who had adrenalectomies and unilateral disease with postoperative confirmation of biochemical cure (normal aldosterone and plasma renin activity), (Figure 1). We analyzed demographics, systolic and diastolic blood pressure, serum aldosterone and plasma renin level, potassium level, perioperative clinical data, and AVS results [20]. All patients had confirmatory testing for PA with either a sodium chloride loading test, a captopril test, or a posture test. The median follow-up duration was 21 months (range 1–184). Data were obtained from a prospective study protocol approved by our Institutional Review Board in 2019 (NCI-09-C-0242). All patients provided written informed consent.

### 2.2. AVS Technique

Each AVS was performed by an experienced interventional radiologist. The patients underwent simultaneous bilateral femoral venous catheterization followed by bilateral adrenal venous catheterization. Simultaneous venous blood samplings from the right and left adrenal veins—and the right iliac vein for peripheral samples—were obtained. Baseline blood samples were drawn without ACTH stimulation and after an intravenous bolus of 0.25 mg of ACTH followed by continuous infusion of ACTH (0.25 mg in 250 mL normal saline) [20]. Blood samples for post-ACTH infusion were collected 5, 10, and 15 min post-infusion. Adrenalectomy was recommended for all patients who lateralized on AVS with and/or without ACTH stimulation. The selectivity index (SI, cut-off value ≥2) was used for confirmation of successful cannulation of the adrenal vein. The lateralization index (LI, cut-off value >2 and >4) was used for distinguishing between unilateral and bilateral PA [21].

### 2.3. Statistical Analysis

Continuous variables are presented as mean ± standard deviation or as medians and ranges. Categorical variables are presented as absolute numbers and percentages. Comparisons between groups were assessed with the chi-square test or Fisher’s exact test for categorical variables, and the *t-*test or the Mann–Whitney U test for continuous variables. All data were analyzed using SAS version 9.4 (SPSS Inc., Chicago, IL, USA). A *p* value <0.05 was considered statistically significant.

## 3. Results

A total of 76 patients had adrenalectomies for unilateral PA, and the demographic and clinical characteristics of the study cohort are summarized in Table 1. The median age was 52 years, with a median history of hypertension of 12 years. Among the 76 patients, 74 lateralized using LI > 2 and 72 lateralized using LI > 4 with and/or without ACTH stimulation. (Figure 1) All patients who had an adrenalectomy had a biochemical cure based on normal postoperative measurements of serum aldosterone and plasma renin activity levels. According to the Primary Aldosteronism Surgical Outcomes criteria, complete and partial success were observed in 26 (34%) and 50 (66%) patients, respectively [22].

The mean SI for the right and left adrenal vein was 41.7 and 33.2 with ACTH stimulation and 5.8 and 4.9 without ACTH stimulation, respectively (Table 2). The SI ratio was significantly higher with ACTH stimulation compared to without ACTH stimulation for the right adrenal vein (93.4% vs. 50.0%, *p* = 0.027). For the left adrenal vein, the SI ratio was higher with ACTH stimulation compared to without (97.4% vs. 59.2%) with no statistically significant difference (*p* = 0.084). The mean LI was 34.7 with ACTH stimulation and 19.6 without ACTH stimulation (Table 3). The LI > 2 ratio was significantly higher with ACTH stimulation compared to without (97.4% and 90.8%, respectively, *p* = 0.007). However, there was no statistically significant difference when using LI > 4 (85.5% vs. 76.3%, *p* = 0.239). In the 31 patients who did not have an operation, using a cutoff of LI > 2 and >4, 4 (12.9%) and 3 (9.6%) were lateralized without ACTH stimulation, respectively, but not lateralized with ACTH stimulation.

The subset of LI proportion was compared with and without ACTH stimulation, using the cut-off values of >2 and >4 (Table 4). With LI > 2, 69 (90.8%) patients were lateralized both with and without ACTH stimulation. There were 5 patients (6.6%) who had lateralization with but not without ACTH stimulation. With LI > 4, 51 (67.1%) patients were lateralized both with and without ACTH stimulation. Fourteen (18.4%) patients were lateralized with ACTH stimulation alone. However, there were 7 patients (9.2%) who only lateralized without ACTH stimulation.

## 4. Discussion

AVS is recommended to distinguish between unilateral and bilateral PA before adrenalectomy [2,6,7,8,10,11,23]. However, there is still some debate about performing routine versus selective AVS in all patients with PA. AVS is considered safe to perform: it has around a 0.7% complication rate of adrenal vein rupture [7,17,24]. The procedure clearly holds the benefit of identifying surgically curable PA under experienced physicians. In our previous study, we stated that AVS is critical for appropriately selecting patients who have unilateral disease and who would benefit from adrenalectomy. We found that the specific technique and criteria for determining lateralization, based on AVS results, is important [20].

Some studies have highlighted the lack of added value in performing AVS in patients with unilateral adrenal masses and normal contralateral adrenal glands on sensitive anatomic imaging studies. An important shortcoming of AVS is that the result may vary depending on the experience of the interventional radiologist who performs the procedure [25].

Based on current clinical guidelines, the majority of centers perform AVS before considering patients as candidates for adrenalectomy [6,10,15]. AVS can be performed with and/or without ACTH stimulation [2,8,15,16,17,18]. However, the optimal protocol guidelines are not clear with regard to the administration of ACTH stimulation. ACTH stimulation is preferred due to certain advantages compared to not performing it [8]. ACTH stimulation avoids the problem of aldosterone and cortisol pulsatile variation and increase cortisol secretion that results in a greater gradient compared to non-stimulation. Lim et al. reported that AVS performed via continuous ACTH stimulation resulted in a 95.5% effective surgical cure rate for PA [26]. When ACTH stimulation is used, most studies have validated that an LI > 4 may be appropriate for determining unilateral disease in PA [2,21,22]. Other experts do not believe ACTH stimulation is helpful [27,28]. AVS results with ACTH stimulation presented no consistency for identifying surgically curable PA [15]. In certain studies, AVS with and without ACTH stimulation have both been recommended in distinguishing surgically curable PA [18,27].

In this study, the same patient had simultaneous cannulation of bilateral adrenal veins as well as AVS with and without ACTH stimulation. Not many studies have data on with and without ACTH stimulation, simultaneously in the same patient, to provide how the results can be different in both groups when certain LI cutoffs are used. We included patients who had adrenalectomies only, since post-adrenalectomy status can show if they were biochemically cured and that they really had unilateral PA. This study revealed that AVS with ACTH stimulation is associated with a higher SI for the right and left adrenal vein compared to AVS without ACTH stimulation. For both right and left adrenal veins, SI ≥ 2 ratios were higher for ACTH stimulation compared to no ACTH stimulation (93.4% and 97.4% vs. 50% and 59.2%, respectively). This finding is consistent with a recent study showing that ACTH stimulation improves the SI of AVS [18]. Deinum et al. reported that ACTH stimulation during AVS is related to high SI values and emphasized that besides SI, demonstrating lateralization is more important when performing AVS because the purpose of AVS is to distinguish unilateral from bilateral disease [15]. However, their study showed AVS with ACTH stimulation did not present consistent LI. Some investigators have found that the LI decreases with ACTH stimulation, in contrast to our data [29]. Some have speculated this is due to ACTH receptor expression in cortical adenoma may vary depending on ion channel mutation present or that adjacent nodular hyperplasia may respond more than the adenoma.

When LI > 2 was applied, the LI ratio was significantly higher with ACTH stimulation (97.4%) compared to without ACTH stimulation (90.8%) in patients with PA (*p* = 0.007). However, with LI > 4, the LI ratio was higher with ACTH stimulation compared to without, but the difference was not statistically significant (*p* = 0.239). Although these results suggest that ACTH stimulation presents a higher SI, the benefit of using only ACTH stimulation to lateralize PA patients is still unclear when a LI > 4 is used to determine candidates for adrenalectomy [15,21].

We analyzed the subset of patients that fall in the category of ≤ or >2 and 4, with and without ACTH stimulation. We found that 9.2% of patients with PA did not lateralize with ACTH stimulation but did so without ACTH stimulation, based on LI > 4 [20]. This finding suggests that AVS without ACTH stimulation—in addition to AVS with ACTH stimulation—identifies a greater number of patients with PA and unilateral disease that would be candidates for adrenalectomy when LI > 4 is used. We believe that both with and without ACTH stimulation are useful to identify surgically curable patients. However, if a LI > 2 is used, there were no additional patients who had lateralization without ACTH stimulation.

There are several limitations to our study. Selection bias could be present due to our institution being a referral center and because of the retrospective study design. This means that our cohort may not reflect the majority of patients with PA. However, the strengths of this study are that each patient had a standardized approach for AVS with and without ACTH stimulation, performed in each patient at the same time to determine what would be the optimal approach for AVS and to distinguish unilateral versus bilateral disease. In addition, a single experienced interventional radiologist performed all of the AVSs to present uniform results with an adrenal vein cannulation rate of greater than 98%.

In summary, we found that AVS with ACTH stimulation was associated with a higher SI ratio and LI > 2 ratio. However, the LI ratio did not differ with and without ACTH stimulation when LI > 4 was used. In fact, using LI > 4 with ACTH stimulation, 9.2% of the patients could have been omitted even though they were candidates for surgically curable PA who lateralized without ACTH stimulation. For this reason, we believe that a strategy with and without ACTH stimulation is useful during AVS to identify a greater number of patients who could be candidates for adrenalectomy when LI > 4 is used. In conclusion, without ACTH stimulation should also be included because it detects a subset of patient with unilateral disease that could be considered for adrenalectomy but are not detected with ACTH stimulation.

## Figures and Tables

**Table 1:** Demographic and clinical characteristics of the study cohort.

	Distribution, Median (Range)
Sex	
Male	46
Female	30
Age (Years)	52 (23–72)
History of Hypertension (Years)	12 (0.3–40)
Serum K^+^ (mEq/L)	3.2 (1.9–4.6)
Serum Aldosterone (ng/dL)	35.0 (2.0–194)
Serum Renin (ng/mL/hour)	0.6 (0.1–3.5)
Serum Creatinine (mg/dL)	1.0 (0.5–2.3)
Body Mass Index	30 (19.0–47.7)
Number of Anti-Hypertensive Medications	3 (0–7)
Cardiovascular Disease History	22
Adrenalectomies	76

**Table 2:** Selectivity index results in 76 patients with and without adrenocorticotropic hormone (ACTH) stimulation.

Selectivity Index	With ACTH Stimulation	Without ACTH Stimulation	*p*
Mean (SD)			
Right side	41.7 (27.4)	5.8 (11.3)	<0.001
Left side	33.2 (18.8)	4.9 (6.3)	<0.001
Cut-off value ≥ 2			
Right side	71 (93.4%)	38 (50.0%)	0.027
Left side	74 (97.4%)	45 (59.2%)	0.084

SD, standard deviation.

**Table 3:** Lateralization index results in 76 patients with and without adrenocorticotropic hormone (ACTH) stimulation.

Lateralization Index	With ACTH Stimulation	Without ACTH Stimulation	*p*
Mean (SD)	34.7 (59.3)	19.6 (25.0)	<0.001
Cut-off Value >2	74 (97.4%)	69 (90.8%)	0.007
Cut-off Value >4	65 (85.5%)	58 (76.3%)	0.239

SD, standard deviation.

**Table 4:** Lateralization index comparison in 76 patients with and without adrenocorticotropic hormone (ACTH) stimulation.

Lateralization Index		With ACTH stimulation			
		≤2	>2	≤4	>4
**Without ACTH Stimulation**	≤2	2 (2.6%)	5 (6.6%)		
	>2	0	69 (90.8%)		
	≤4			4 (5.3%)	14 (18.4%)
	>4			7 (9.2%)	51 (67.1%)
